# A Review of Extracellular Vesicles in COVID‐19 Diagnosis, Treatment, and Prevention

**DOI:** 10.1002/advs.202206095

**Published:** 2023-05-05

**Authors:** Peng Su, Yuchen Wu, Feng Xie, Qinghui Zheng, Long Chen, Zhuang Liu, Xuli Meng, Fangfang Zhou, Long Zhang

**Affiliations:** ^1^ Department of Breast Surgery Zhejiang Provincial People's Hospital Hangzhou 310014 P. R. China; ^2^ Institutes of Biology and Medical Science Soochow University Suzhou 215123 P. R. China; ^3^ Department of Clinical Medicine The First School of Medicine Wenzhou Medical University Wenzhou Zhejiang 325035 P. R. China; ^4^ Center for Translational Medicine The Affiliated Zhangjiagang Hospital of Soochow University Zhangjiagang Jiangsu 215600 China; ^5^ Institute of Functional Nano and Soft Materials (FUNSOM) Jiangsu Key Laboratory for Carbon‐Based Functional Materials and Devices Soochow University Suzhou Jiangsu 215123 China; ^6^ MOE Laboratory of Biosystems Homeostasis & Protection and Innovation Center for Cell Signaling Network Life Sciences Institute Zhejiang University Hangzhou 310058 P. R. China; ^7^ Cancer Center Zhejiang University Hangzhou Zhejiang 310058 P. R. China

**Keywords:** coronavirus disease 2019, diagnosis, drug delivery, extracellular vesicles, neutralization traps

## Abstract

The 2019 novel coronavirus disease (COVID‐19) pandemic caused by severe acute respiratory syndrome coronavirus 2 (SARS‐CoV‐2) is ongoing, and has necessitated scientific efforts in disease diagnosis, treatment, and prevention. Interestingly, extracellular vesicles (EVs) have been crucial in these developments. EVs are a collection of various nanovesicles which are delimited by a lipid bilayer. They are enriched in proteins, nucleic acids, lipids, and metabolites, and naturally released from different cells. Their natural material transport properties, inherent long‐term recycling ability, excellent biocompatibility, editable targeting, and inheritance of parental cell properties make EVs one of the most promising next‐generation drug delivery nanocarriers and active biologics. During the COVID‐19 pandemic, many efforts have been made to exploit the payload of natural EVs for the treatment of COVID‐19. Furthermore, strategies that use engineered EVs to manufacture vaccines and neutralization traps have produced excellent efficacy in animal experiments and clinical trials. Here, the recent literature on the application of EVs in COVID‐19 diagnosis, treatment, damage repair, and prevention is reviewed. And the therapeutic value, application strategies, safety, and biotoxicity in the production and clinical applications of EV agents for COVID‐19 treatment, as well as inspiration for using EVs to block and eliminate novel viruses are discussed.

## Graphical abstract

A variety of engineered Angiotensin‐Converting Enzyme 2‐extracellular vesicles (ACE2‐EVs) with different properties have been developed. Different methods are used, including increasing the membrane ACE2 level and neutralization efficiency of EVs using palmitoylation modification or CD9 scaffolds, fusing THP‐1‐derived EVs and ACE2‐EVs to neutralize both SARS‐CoV‐2 and cytokins to alleviate the inflammatory storm.

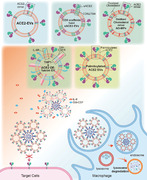

## 1 Introduction

The coronavirus disease 2019 (COVID‐19) pandemic caused by a novel severe acute respiratory syndrome coronavirus 2 (SARS‐CoV‐2) has resulted in over 537 million confirmed cases and 6.3 million deaths worldwide in the past two and a half years.^[^ ^1^, ^2^ ^]^ Although this significant public health emergency has been mitigated via vaccination of the population using multiple vaccines and the development of treatment strategies, currently, approximately 300 000 new cases remain daily (data from WHO COVID‐19 Dashboard. Available online: https://covid19.who.int/. last cited: 12th, Sep, 2022). This indicates that the novel coronavirus continues to pose a persistent threat to public health and safety, as its high transmission capacity, high mutation frequency, and evolving immune escape potential. Importantly, this pandemic has increased our awareness of the continued need to develop new, mature, and well‐established blocking, diagnostic, and therapeutic systems that effectively control pandemic progression in the early stage of an epidemic in which new unknown viruses emerge and spread. Extracellular vesicles (EVs), with their excellent biological characteristics and malleability, are one of the most promising means for the above challenges.

EVs are widely recognized as a general term for vesicular granules which are naturally released by all cells, including prokaryotic and eukaryotic cells. They are delimited by a lipid bilayer and are replication inactive. EVs can be broadly classified into two subtypes, ectosomes and exosomes, based on their biogenesis. Ectosomes refer to vesicles formed by the outward budding of the plasma membrane that exists in a wide range of diameters between ≈50 nm and 1 µm, also known as microvesicles or microparticles. The biogenesis of exosomes is relatively complex. The plasma membrane is sequentially invaginated to form endosomes and multivesicular bodies (MVB), and then the outer membrane of the MVBs fuses with the plasma membrane to release the exosomes. Due to this mechanism, the sizes of the exosomes are strictly limited between ≈40 and 160 nm. Unfortunately, the current isolation and purification methods used by laboratories and commercial companies are primarily based on the size or density of the EVs, including size‐exclusion chromatography, density gradient centrifugation, and ultrafast differential centrifugation, or based on the biochemical composition of EVs, such as anti‐CD63/CD81 immune isolation. These strategies fail to effectively separate the ectosomes and exosomes based on their distinct biogenesis, and studies providing sufficient stable and reliable biomarkers for the technical separation of these two subtypes are unavailable. According to the proposal in Minimal Information for Studies of EVs 2018 (MISEV2018), extracellular particles isolated and purified by non‐specific methods are generically referred to as EVs, unless labeled by a specific marker of subcellular origin. The EVs described in this review also mainly refer to small EVs with diameters between ≈50 and 200 nm.

In recent years, EVs have gained attention because of their high abundance in human internal environments and bodily fluids, as well as the diversity and plasticity of their cargos, including parent cell‐derived DNA, RNA, lipids, metabolites, and cytosolic and cell‐surface proteins. Increasing research demonstrates their important pathogenic role and modulatory capabilities in immune inflammation, cancer, cardiovascular disease, and neurodegenerative diseases. For instance, EVs enriched with programmed death‐ligand 1 (PD‐L1) from highly metastatic cancer cells are secreted into the host peripheral circulatory system, directly contacting peripheral CD8 T cells to suppress systemic T cell immunity and facilitate distal metastasis.^[^ ^3^, ^4^ ^]^ miR‐122 in breast cancer cell‐derived EVs targets pyruvate kinase M1/2 (PKM) in beta cells, inhibiting glycolysis and ATP‐dependent insulin secretion, disrupting host glucose metabolism, and promoting tumor growth.^[^ ^5^, ^6^ ^]^ Additionally, the natural therapeutic potential of EVs can be exploited. Mesenchymal stem cell (MSC)‐derived EVs (MSC‐EVs) can alleviate CCl4‐induced ferroptosis and acute liver injury by downregulating peroxidation levels of liver lipids and restoring SLC7A11 protein levels in hepatocytes.^[^ ^7^ ^]^ Foxp3+ Treg cell‐derived EVs can suppress immune rejection after in vivo transplantation due to the presence of neuropilin‐1 on the surface of EVs.^[^ ^8^ ^]^ Furthermore, Kupffer cells (KCs) deliver endogenous miR‐690 to other hepatocytes via EVs, inhibiting de novo lipogenesis in hepatocytes, fibrogenesis in hepatic stellate cells, and inflammation in recruited hepatic macrophages, suggesting that KC‐EVs may be promising therapeutics for nonalcoholic steatohepatitis.^[^ ^9^ ^]^ During the COVID‐19 pandemic, EV‐based diagnosis and therapeutic tools are important. In this review, we provide basic information on EVs and highlight the latest advances in EV‐based diagnosis and therapeutic applications in COVID‐19 and its complications.

## 2 Biogenesis, Purification, and Composition of EVs

**Figure 1:**
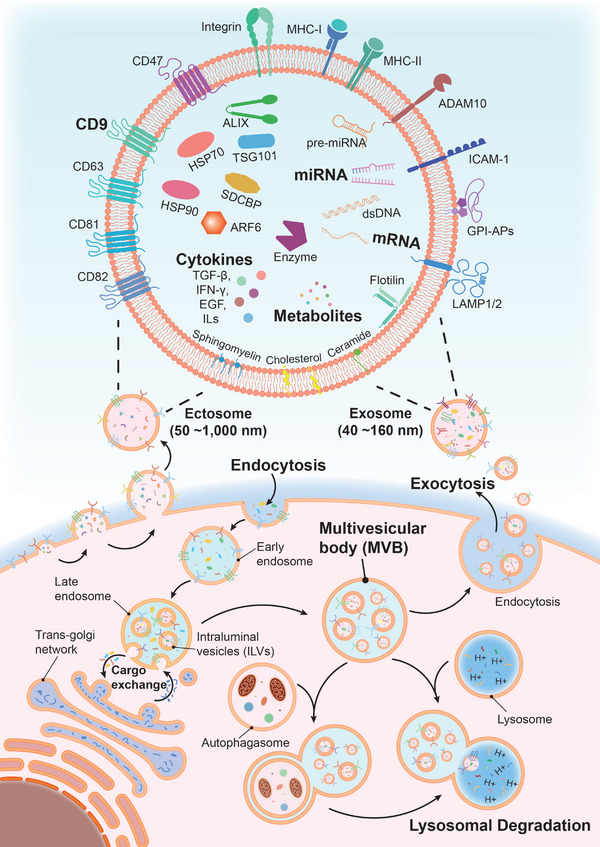
Molecular composition, biogenesis, and secretion of extracellular vesicles (EVs). EVs contain complex contents including proteins, nucleic acids, lipids, and metabolites. Some membrane proteins, such as CD9, and encapsulated microRNAs (miRNAs), mRNAs, cytokines, and metabolites are currently utilized for clinical therapeutic tasks. EVs consist of two major subgroups: ectosomes and exosomes. Ectosomes are formed by direct budding of the cytomembrane. Exosomes undergo endocytosis at the cytoplasmic membrane, secondary endocytosis at the endosomal membrane, formation of multivesicular bodies (MVBs), and finally follow steps for degradation or release. The process involves the exchange of cargo with other intracellular vesicular substructures. The released EVs deliver information to recipient cells. Thus, EVs function as a mode of intercellular communication and molecular transfer.

Two pathways of EV formation are generally accepted to occur simultaneously in host cells under physiological conditions (**Figure** **1**). The first is outward budding and fission of the plasma membrane to form ectosomes.^[^ ^10^, ^11^ ^]^ The second is two‐step endocytosis of the plasma membrane to form MVBs, which then fuse with the plasma membrane to release exosomes.^[^ ^12^, ^13^, ^14^, ^15^ ^]^

The formation of ectosomes is generally initiated by a composition change in the local cytoplasmic membrane. The expression of some kinases or release of certain second messengers (e.g., Ca2+) generates a series of cascade signals and activates phospholipid translocators, such as floppases and scramblases, at the cell membrane surface. This results in the translocation of phosphatidylserine to the cell membrane surface, clustering and alteration of transmembrane proteins via interaction or oligomerization potential, or aggregation of other membrane lipids (such as cholesterol) on the membrane.^[^ ^16^, ^17^, ^18^ ^]^ These results may lead to the asymmetry of membrane lipids and local membrane curvature. Subsequent actomyosin contraction assists in the further local membrane budding, and the actin‐myosin‐based contraction at the neck of the ectosome completes the formation and separation of the ectosome.^[^ ^19^, ^20^, ^21^ ^]^ Actin‐myosin plays an integral role in the formation of the ectosome,^[^ ^22^, ^23^, ^24^ ^]^ and ADP‐ribosylation factor 6 (ARF6) is also important because it extensively regulates actin‐myosin remodeling at the cell periphery and endosomal recycling.^[^ ^25^, ^26^ ^]^ ARF6, a member of the small G protein subfamily, activates RhoA (a Rho GTPase),^[^ ^27^ ^]^ which not only phosphorylates myosin light‐chain (MLC) to promote myosin contraction, but also activates RHO‐associated protein kinase and phosphorylates Lim kinase to prevent actin cleavage via cofilin activation.^[^ ^28^ ^]^ Furthermore, ARF6 also can activate phospholipase D to recruit ERK and activate MLC kinase, followed by promoting actomyosin contraction and ectosome release.^[^ ^21^ ^]^

The biogenesis of exosomes is relatively complex and typically involves the crosstalk of intracellular vesicle transport. The uptake of non‐transmembrane transported cargos in the extracellular environment or ligand‐receptor binding on the cell membrane triggers physiological endocytosis of the cytoplasmic membrane and initiates the formation of early endosomes,^[^ ^29^ ^]^ which is the first endocytosis step required for exosome biogenesis.^[^ ^30^, ^31^ ^]^ Some early endosomes undergo a second endocytosis step as they mature into late endosomes and encapsulate cargo from the cytoplasm, trans‐Golgi network, or other intracellular vesicles into intraluminal vesicles (ILVs) and form characteristic MVBs.^[^ ^31^, ^32^, ^33^, ^34^ ^]^ The MVBs then have two potential fates: fusing with autophagic vesicles or lysosomes to reach lysosomal degradation, or fusing with the plasma membrane and releasing ILVs as exosomes outside the cell.^[^ ^12^, ^15^, ^35^, ^36^ ^]^ The two distinct fates of MVBs are strictly governed by known but incomplete molecular mechanisms. As such, the chemotactic capacity of MVBs is based on microtubule networks and molecular motors. The small GTPase Rab7 on the membrane of MVBs interacts with lysosomal proteins and then binds with the p150 subunit of the dynactin protein complex, a retrograde molecular motor, thereby retrogradely transporting MVBs along microtubules to lysosomes, which may be prevented by high cholesterol conditions.^[^ ^15^, ^37^ ^]^ Cholesterol‐rich MVBs are more inclined to exosome secretion.^[^ ^38^ ^]^ Conversely, overexpression of K38R‐Rab7 or depletion of the E3 ubiquitin ligase Parkin promotes exosome secretion, suggesting that Rab7 without the K38‐site ubiquitination modification in turn assists MVBs for exosome secretion.^[^ ^39^ ^]^ Rab27a and Rab27b are more suitable markers for the exosome release pathway of MVBs because Rab27a primarily mediates the docking of MVBs to the plasma membrane and rearrangement of the sub‐membrane actin cytoskeleton, whereas Rab27b translocates MVBs from microtubules to the cell periphery.^[^ ^40^, ^41^, ^42^ ^]^

Moreover, the process by which late endosomes undergo secondary endocytosis to form MVBs has been extensively studied. The endosomal sorting complex required for transport (ESCRT) machinery was first identified to drive secondary endocytosis.^[^ ^43^ ^]^ ESCRT‐0 (as known as HRS or HGS) and ESCRT‐I aggregate the ubiquitinated membrane proteins and recruit the ESCRT‐II/ESCRT‐III complex to perform budding and fission of the microdomain.^[^ ^44^, ^45^, ^46^ ^]^ Other cargos, such as nucleic acids and cytoplasmic proteins, are captured by the syndecan‐syntenin‐ALIX (ESCRT accessory protein ALG‐2 interacting protein X) axis and bind to the VSP32 subunit of the ESCRT‐III complex, which is eventually transported into the ILVs of MVBs.^[^ ^47^ ^]^ However, cells may still form and secrete exosomes despite ESCRT complex depletion, which is mainly attributed to ceramide and tetraspanins.^[^ ^48^, ^49^ ^]^ Ceramides, derived from the hydrolysis of sphingolipids, are enriched in microdomains and induce a natural negative curvature to drive the budding of the late endosomal membrane.^[^ ^49^, ^50^ ^]^ Tetraspanins, such as CD63, CD81, and CD9, are also involved in cargo sorting and microdomain formation through their oligomerization,^[^ ^51^, ^52^, ^53^, ^54^ ^]^ and cholesterol further promotes microdomain budding by entering the conical structural cavities of these tetraspanins.^[^ ^55^ ^]^

Absolute purification and separation of ectosomes and exosomes are not yet possible because of their distinct biogenesis. However, mixed samples (containing ectosome, exosome, and other vesicles collectively referred to as EVs) isolated by commonly used separation and concentration techniques are currently available for most basic research and clinical applications. According to the statistics of MISEV2018, differential ultracentrifugation (dUC) is the most common primary EV separation and concentration technique.^[^ ^56^ ^]^ The broad steps include: 1) low‐speed centrifugation at 500 g for 10 min to remove suspended cells from the sample suspension; 2) centrifugation at 2000 g for 20 min to discard dead cells; 3) high‐speed centrifugation at 10 000 g for 1 h to eliminate large microvesicles and cellular debris (generally, ectosomes with diameters between ≈300 nm and 1 µm and other extracellular microvesicles are more enriched in the precipitate in this step); 4) ultracentrifugation at 120 000 g for 70 min to concentrate the small EVs (sEV) in a small suspension at the bottom of the ultracentrifuge tube; 5) removal of the upper suspension and resuspension of the sEV‐rich bottom suspension with a sufficient amount of 0.22 µm filtered phosphate‐buffered saline (PBS) to reduce the overall lipid solubility of the suspension; and 6) centrifugation at 120 000 g for 70 min to precipitate sEVs at the bottom of the ultracentrifuge tube. The sEV‐concentrated suspension is obtained by gently blowing the suspension with little PBS.^[^ ^57^, ^58^, ^59^ ^]^ Density gradient centrifugation (dgUC) can isolate EVs with higher purity than dUC. The steps can be broadly divided into the following: 1) initial centrifugation of the sample to remove dead cells and cell debris by centrifugation at 2000 and 10 000 g; 2) prepare a density gradient solution by mixing the appropriate amount of sucrose or iodixanol with a buffer (e.g., PBS) to form a gradient ranging from low to high density (e.g., 10–60%); 3) Layer the sample on top of the density gradient solution and centrifuge at a high speed (e.g., 100 000 g) for at least 2–3 h (or until the gradient is fully formed); 4) carefully collect fractions and buffer systems located in each density interval from the density gradient interface using a needle and syringe or pipette; 5) take a small sample from each interval fraction, and identify the various markers of EVs (such as ALIX, TSG101, CD63) through Western blotting to determine the optimal density range for EVs. EVs are typically enriched in the density range of 1.10 to 1.18 g mL^−1^. Although dgUC separation provides higher purity of EVs, it requires higher operational requirements and has limited sample loading capacity, which cannot meet the demand for rapid large‐scale isolation and purification.^[^ ^57^, ^58^, ^60^, ^61^ ^]^ Precipitation based on the addition of polymers to alter the solubility of EVs allows the isolation and purification of EVs on a large scale without expensive ultracentrifugation equipment. First, add a polymer solution (e.g., PEG 6000 or 8000) to the sample to achieve a final concentration of 5%–10%. Then, incubate the sample on ice or at 4 °C for 30–60 min. Finally, centrifuge the sample at a low speed (e.g., 3000 g) for 20–30 min, discard the supernatant, and resuspend the EV‐containing pellet in an appropriate buffer. Although this method is simple and fast, the obtained product often contains a large amount of co‐precipitated impurities such as proteins. Researchers should consider this method according to the subsequent experimental requirements.^[^ ^58^, ^60^, ^62^, ^63^ ^]^ Ultrafiltration and size‐exclusion chromatography (SEC) both perform the separation and purification of EVs based on the physical radius of EVs. Ultrafiltration requires different pore sizes of nanomembranes or molecular weight cut‐off (MWCO) membranes suitable for the sample type. After the sample passes through the filtration system, large molecular impurities such as proteins are filtered out while EVs are retained on the membrane or filter. Ultrafiltration separation greatly shortens the centrifugation time, but membrane clogging can lead to loss and mechanical damage of EVs. This requires researchers to establish a reasonable ultrafiltration separation strategy and monitor the membrane pressure in a timely manner.^[^ ^58^, ^60^, ^62^, ^63^ ^]^ On the other hand, SEC requires the experimenter to pack a column with a porous matrix (e.g., Sepharose CL‐2B or Sephadex G‐100) and equilibrate with an appropriate buffer (e.g., PBS, Tris‐HCl). Then the sample is loaded onto the column and flows through the column gently and slowly under gravity or low pressure. Collecting fractions that flow out at different time points, EVs are determined by monitoring the absorbance at 280 nm or by analyzing EVs markers.^[^ ^57^, ^58^, ^60^, ^64^, ^65^ ^]^ Different from these above separation methods based on the physical properties of EVs, immunoaffinity capture utilizes the interaction between specific antibodies and EVs membrane protein markers (such as CD81, CD63, CD9) to obtain high‐purity EVs and specific EV subpopulations. After incubating the sample with the coupled specific antibodies on magnetic beads or chromatography matrix, the contained EVs will be enriched on the solid‐phase matrix, and other non‐specific impurities will be removed after multiple washes. Finally, the EVs are eluted by adding peptides with stronger competitive binding ability. It should be noted that when selecting specific antibodies for the isolation and separation of EV subpopulations, the membrane protein characteristics of the target EV subpopulation and the sample source should be fully considered. The comparison between different isolation methods is shown in **Table** **1**.^[^ ^57^, ^58^, ^59^, ^60^, ^61^, ^62^, ^63^, ^64^, ^65^ ^]^ Moreover, increasing combinations of techniques and separation technologies are gradually being developed, such as asymmetric flow field‐flow fractionation,^[^ ^66^, ^67^ ^]^ alternating current electrophoresis,^[^ ^68^, ^69^ ^]^ ion exchange chromatography,^[^ ^70^, ^71^ ^]^ deterministic lateral displacement arrays,^[^ ^72^ ^]^ lipid affinity isolation technologies,^[^ ^73^ ^]^ and several microfluidic systems based on combinations of these principles.^[^ ^74^, ^75^, ^76^ ^]^ All exhibit some potential to meet the demands of sEV separation and purification with high throughput, high recovery, and high specificity.

**Table 1:** The different isolation processing techniques of extracellular vesicles and their advantages/disadvantages/applicability

Isolation methods	Principle	Advantages	Disadvantages	Recovery rate	Purity	Applicability
Differential Ultracentrifugation (dUC)	Separation based on size, density among EVs and other biomolecules	Large sample capacity, Easy to operate, Low cost, No additional chemicals, Well‐established method	Time‐consuming, Requires specialized equipment, Possible mechanical damage of EVs, Impurities (e.g., protein aggregates)	Medium	Medium	EVs from various biological fluids
Density Gradient Ultracentrifugation (dgUC)	Separation based on density using a gradient medium	High purity, No additional chemicals, Well‐established method	Time‐consuming for preparation, Low throughput, Requires specialized equipment, contamination of density gradient medium	High	High	Isolation of highly purified EVs
Polymer‐Based Precipitation	Alter the solubility or dispersibility of EVs by adding synthetic polymers	Easy to operate; High efficiency and Low timing‐cost, Gentle method, no specialized equipment needed	Containing co‐precipitation and non‐EV contaminants, Need to remove the crowding agent	High	Low	Large‐scale isolation of EVs with low purity requirements
Ultrafiltration	Separation based on size of EVs and other biomolecules, concentration of EVs using defined pore size membranes	Simple procedure, Time efficient, Relatively gentle, No additional chemicals, No special equipment required	Prone to membrane clogging, resulting in reduced yield, loss, and deformation of EV	Medium	Low	Concentration of EVs, combined with other methods for EV isolation
Size‐Exclusion Chromatography (SEC)	Separation based on differences in hydrodynamic radius of EVs and other biomolecules	High purity and efficiency, Reproducibility, Minimise co‐isolation of contaminants	Expensive instruments and columns, Lengthy process, Low yield	Medium	High	EVs from biological fluids containing large amounts of protein aggregates
Immunoaffinity capture	Specific capture of EVs using antibodies for anti‐EV membrane proteins	High specificity, allows for the isolation of EV subtypes	High reagent cost and Low yield, Lack of universal EVs markers and commercial antibodies	Low	High	Isolation of EV subtypes based on surface markers
Microfluidics	Microscale flow manipulation based on size, shape, and surface biochemical characteristics	High throughput, Fast and Efficient, Low consumable requirements, Easy to automate	Limited sample loading, Risk of pipe clogging, Need for standardization	High	High	Isolation of EV subtypes, high‐throughput screening

The cargos of EVs mainly comprise proteins, nucleic acids, lipids, and other cellular metabolites (Figure 1). According to the Vesiclepedia and Exocarta databases, approximately 350 000 proteins, 38 000 RNAs (including mRNA, microRNA [miRNA], and long non‐coding RNA [lncRNA]), and over 600 lipids and metabolites have been currently identified in EVs. These EVs are significantly heterogeneous based on their different sources (cell culture supernatants, liquid biopsy samples). However, as biogenesis is similar across different EVs, there are still multiple groups of cargos that can be identified in most EVs. This includes the tetraspanins CD63, CD81, CD82, and CD9; ESCRT‐machinery‐related proteins TSG101 (a core component of the ESCRT‐I complex), syntenin (SDCBP), ALIX, and VPS4A/B; the “don't eat me” signaling molecule CD47; major histocompatibility complex I (MHC‐I); heat shock proteins HSP70 and HSP90; cytoskeletal proteins actin and tubulin; ARF6, intracellular adhesion molecule 1 (ICAM‐1), integrins, lysosome‐associated membrane protein (LAMP1/2), and disintegrin and metalloproteinase domain‐containing protein 10 (ADAM10); and some cytokines including transforming growth factor‐beta (TGF‐*β*), interferon‐gamma (IFN‐*γ*), vascular endothelial growth factor (VEGF) A, epidermal growth factor (EGF), and interleukins (ILs). Furthermore, EVs contain several nucleic acids, primarily mRNA, miRNA, and lncRNA. EV‐miRNAs have been relatively more studied compared with other nucleic acids of EVs because of their specific functions derived from their host cells. For instance, EV‐miRNAs derived from tumor cells often promote tumor growth and metastasis by suppressing host immunity, inducing reprogramming in epithelial‐mesenchymal transition (EMT), or impairing metabolic homeostasis.^[^ ^6^, ^77^, ^78^, ^79^, ^80^, ^81^, ^82^, ^83^, ^84^, ^85^, ^86^ ^]^ In contrast, miRNAs enriched in MSC‐EVs possess various therapeutic potentials derived from MSCs, including the amelioration of adverse inflammatory responses and the promotion of tissue repair and regeneration.^[^ ^87^, ^88^, ^89^, ^90^, ^91^, ^92^, ^93^, ^94^ ^]^ The lncRNA BCRT1 in breast cancer derived‐EVs also promotes M2 polarization of tumor‐associated macrophages and hypoxia‐induced EMT in tumor cells, thus promoting breast cancer development and metastasis.^[^ ^95^ ^]^ Moreover, the presence of lipids in EVs cannot be ignored. These lipids mainly include cholesterol, sphingomyelin, ceramide, and phosphatidyl‐choline/ethanolamine/inositol,^[^ ^96^, ^97^, ^98^ ^]^ and the comparative analysis of EV lipidomics is informative and helpful for determining the physiological state of the host and diagnosing diseases.^[^ ^99^, ^100^ ^]^

## 3 EVs as Diagnostic Biomarkers for COVID‐19 and its Complications

A major reason for the rapidly increasing attention on EVs is that their high level in body fluids and abundant cargos suggest that EVs are reliable and convenient diagnostic biomarkers. KRAS mutations in plasma EVs identified in ≈80%–85% of patients with locally advanced and metastatic pancreatic ductal adenocarcinoma (PDAC) may be a broad predictor for cancer‐screening.^[^ ^101^ ^]^ Using next‐generation deep sequencing to analyze the plasma EV‐miRNA expression profiles of patients with Alzheimer's disease (AD), an AD‐specific 16‐miRNA signature is identified to be able to increase the sensitivity and specificity of AD prediction to 87% and 77%, respectively.^[^ ^102^ ^]^

**Figure 2:**
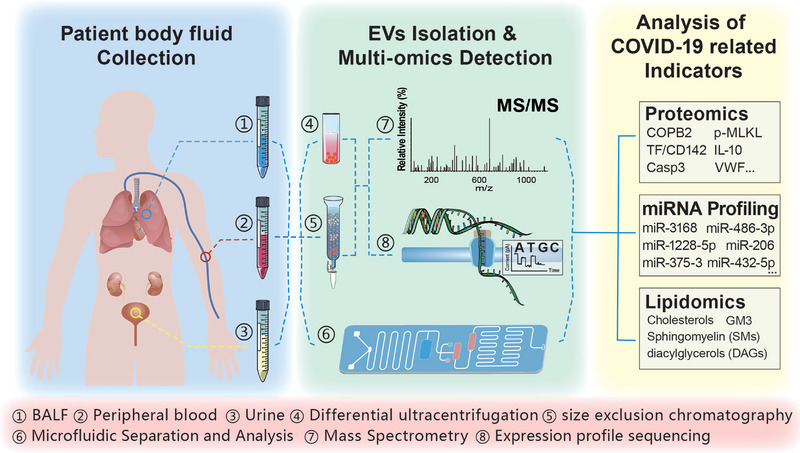
EVs in COVID‐19 diagnosis. Bronchoalveolar lavage fluid (BALF), peripheral blood, and urine from patients with COVID‐19 are collected for fluid biopsy. EVs in the body fluid samples are isolated and purified using differential ultracentrifugation or size‐exclusion chromatography, followed by mass spectrometry and high‐throughput sequencing. Alternatively, EVs in body fluid samples are directly isolated and detected using microfluidic chips. Diagnosis of disease processes and prediction of disease progression are based on multi‐omics data and indicators.

EVs also display high potential in the diagnosis of COVID‐19 and the prediction of mild and severe disease development (**Figure** **2**). SARS‐CoV‐2 infection causes thrombosis and disease severity is highly correlated with the development of thrombosis. Tissue factor (TF)/CD142 plays an important role in hemostasis and thrombosis as a cell surface receptor and cofactor for blood coagulation factors VII and VIIa. Several clinical statistics reveal a significant increase in circulating EV‐TF levels in patients with COVID‐19 and a high correlation with thrombosis, malignant disease progression, and patient hospitalization time.^[^ ^103^, ^104^, ^105^ ^]^ Moreover, proteins involved in inflammation, immune regulation, and tissue remodeling, such as EN‐RAGE and IL‐18R1, are also highly enriched in plasma EVs of patients with COVID‐19 and are positively correlated with disease malignancy, similar to TF.^[^ ^104^ ^]^ In contrast, COPI coat complex subunit beta 2 (COPB2), a subunit of the Golgi coatomer complex, is negatively correlated with COVID‐19 as its level in plasma EVs was significantly higher in patients with mild COVID‐19 than in patients that developed severe/critical COVID‐19 in a discovery cohort, suggesting that patients with high levels of plasma EV COPB2 may be more resistant to disease progression and more likely to experience mild disease.^[^ ^106^ ^]^ Furthermore, platelets directly internalize SARS‐CoV‐2 without angiotensin‐converting enzyme 2 (ACE2) and rapidly exhibit increased apoptosis, necroptosis, distinct morphological changes, and release of EVs. Thus, levels of the apoptotic marker‐activated caspase‐3 and necroptosis marker phospho‐mixed lineage kinase domain‐like pseudokinase (MLKL) in platelet‐derived EVs can be measured to predict the risk of microthrombosis caused by SARS‐CoV‐2.^[^ ^107^ ^]^

Furthermore, miRNAs that are significantly differentially expressed in the circulating EVs of patients with COVID‐19 may also be used as biomarkers to delineate disease risk stratification in patients. Profiling using small RNA sequencing and evaluating using differential gene expression analysis (DGE) indicates 25 significantly downregulated miRNAs (including miR‐375‐3p, miR‐150–5p, miR‐126‐3p, etc.) and 18 significantly increased miRNAs (including miR‐542‐3p miR‐3168, and miR‐1228‐5p) in the plasma EVs of patients with COVID‐19. Compared with those in patients with COVID‐19, 15 significantly upregulated miRNAs (including miR‐486‐3p, miR‐335‐3p, and miR‐432‐5p) and five significantly downregulated miRNAs (including miR‐582‐3p and miR‐206) have been identified in the circulating EVs of critically ill patients who developed acute respiratory distress syndrome (ARDS). The combined analysis allows the use of miRNAs that are highly correlated with the malignant development of COVID‐19, including miR‐3168 and miR‐1228‐5p, as indicators for the diagnosis and prediction of COVID‐19 development.^[^ ^108^ ^]^ Last, the expression levels of EV‐miRNA that target NLRP3 inflammasome, including EV‐miR‐466g and EV‐miR‐466m‐5p, are significantly increased in bronchoalveolar lavage fluid (BALF) to exacerbate the inflammation in mouse ARDS model, suggesting their use as potential predictors for the clinical diagnosis of ARDS development.^[^ ^109^ ^]^

The lipidomic and metabolomic information carried by plasma EVs in patients with mild, moderate, and severe COVID‐19 and healthy controls vary. For example, disease progression of patients with COVID‐19 is accompanied by an increased abundance of monosialodihexosyl ganglioside (GM3) in plasma EVs. Compared with those in healthy controls, circulating EVs in patients with COVID‐19 exhibit increased sphingomyelin (SMs) and reduced diacylglycerols (DAGs),^[^ ^110^ ^]^ as well as significantly dysregulated lipid raft metabolism associated with EVs.^[^ ^99^ ^]^ Although lipidomic and metabolomic analyses of EVs in patients have not yet been included in the list of clinical diagnostic credentials, as the EV isolation and omics investigation techniques continue to mature, the profound host metabolic changes reflected by EVs will be considered.

The development of critical illness in patients with COVID‐19 is characterized by severe immune hyperactivation and endothelial damage.^[^ ^111^, ^112^ ^]^ Based on plasma proteomic analyses and machine learning algorithms, a prominent signature of neutrophil activation, including resistin (RETN), lipocalin‐2 (LCN2), hepatocyte growth factor (HGF), interleukin‐8 (IL‐8), and granulocyte colony‐stimulating factor (G‐CSF) that effectively predict critical illness have been identified.^[^ ^111^ ^]^ Pro‐inflammatory factors, such as IL‐10, that are enriched in macrophage‐derived EVs in BALF further activate neutrophils and promote M2 polarization of macrophages, leading to pulmonary fibrosis.^[^ ^113^ ^]^ Multiple markers associated with endothelial vascular injury caused by SARS‐CoV‐2 infection, such as von Willebrand factor antigen (VWF), plasminogen activator inhibitor‐1 (PAI‐1), and syndecan‐1, are also found in patient thrombosis and plasma.^[^ ^114^ ^]^ These features are highly likely to be enriched in EVs of corresponding origin, which can be used as a basis for disease‐stage diagnosis and critical illness prediction. In addition to the diagnostic applications, MSC‐EVs have also been attempted to treat COVID‐19‐induced inflammatory storms and tissue damage because of their anti‐inflammatory and tissue regeneration abilities inherited from their parent cells.

## 4 MSC‐EVs Prevent the Cytokine Storm and Repair Tissue Damage in COVID‐19

Respiratory and alveolar epithelial cells, particularly type II alveolar epithelial cells, are infected by SARS‐CoV‐2 and hijacked to execute rapid viral expansion because of their high expression of ACE2 and transmembrane protease serine 2 (TMPRSS2).^[^ ^115^, ^116^, ^117^, ^118^, ^119^, ^120^ ^]^ SARS‐CoV‐2 accumulation induces a persistent viral inflammatory response and apoptosis of recruited lymphocytes, thus accentuating the cytokine storm and ultimately triggering ARDS and lung fibrosis injury.^[^ ^121^, ^122^, ^123^, ^124^ ^]^

Moreover, SARS‐CoV‐2 infection of pulmonary capillary endothelial cells not only causes inflammation, platelet aggregation, and fulminant activation of coagulation,^[^ ^125^, ^126^, ^127^, ^128^ ^]^ but also interferes with crucial functions of vascular endothelial cells, including ACE2‐angiotensin (1–7)‐based nitric oxide (NO) synthesis and release, vasodilation, anti‐oxidative stress, and anti‐inflammation, because the spike‐ACE2 interaction reduces ACE2 expression and leads to the endocytosis of membrane ACE2.^[^ ^125^, ^129^, ^130^, ^131^, ^132^ ^]^ These factors contribute to microthrombosis and the high incidence of thrombotic complications, which may eventually induce multiorgan damage and failure.^[^ ^123^, ^133^ ^]^

**Figure 3:**
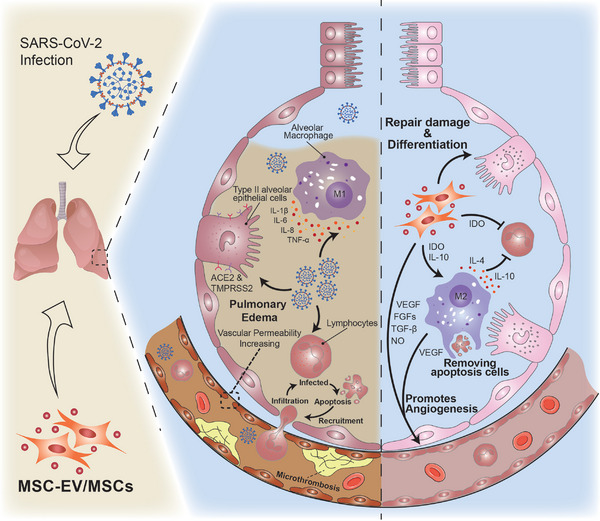
Mesenchymal stem cell (MSC)‐derived EVs (MSC‐EVs) prevent the cytokine storm and repair tissue damage in COVID‐19. SARS‐CoV‐2 infiltrates type 2 alveolar epithelial cells with high expression of angiotensin‐converting enzyme 2 (ACE2) and transmembrane protease, serine 2 (TMPRSS2) and induces M1 polarization of lung macrophages and release of inflammatory factors including IL‐1*β* and IL‐6. Infiltrating lymphocytes are infected by the virus and then apoptosis, which in turn recruits new lymphocytes, creating a vicious cycle of inflammation. Under inflammation, permeability of the blood vessel wall increases, platelets aggregate and form microthrombi, abnormal lung epithelial cell function occurs, and pulmonary edema persists. Infused MSC‐EVs and MSCs repair lung injury and differentiate into type 2 alveolar epithelial cells, inhibit apoptosis, and reduce inflammatory cell infiltration. MSC‐EVs and MSCs also induce M2 polarization of macrophages through indoleamine 2,3‐dioxygenase (IDO) and IL‐10, which enhances their phagocytosis and IL‐10 synthesis to promote the clearance of apoptotic cells. Growth factors, such as VEGF and fibroblast growth factors (FGFs), in MSC‐EVs also promote angiogenesis and diastole, which ultimately clear alveolar fluid and prevent the development of pulmonary fibrosis.

Fortunately, MSCs, which are recognized for their outstanding anti‐inflammatory and tissue repair functions, also present excellent therapeutic potential for the above‐mentioned acute inflammation, ARDS, and tissue and organ damage caused by COVID‐19 (**Figure** **3**). After MSC transplantation (typically intravenous) treatment, patients with severe and critical COVID‐19 show significant improvements in lung function, effective suppression of the cytokine storm, and significantly increased survival.^[^ ^134^, ^135^, ^136^, ^137^ ^]^ Such therapeutic effects are commonly considered to be based on the functions of MSCs, including expression of multiple anti‐inflammatory factors (e.g., IL‐10, G‐CSF, IL‐6R, and CXC chemokines), paracrine secretion of growth factors (including angiopoietin‐1/2, VEGF, and fibroblast growth factor (FGF)), mitochondrial transfer, homing to damaged inflamed tissue, and differentiation potentials.^[^ ^138^, ^139^, ^140^ ^]^ Until now, over 70 clinical trials of MSC therapy for COVID‐19 have been conducted worldwide, including remestemcel‐L, which was approved by the Food and Drug Administration (FDA) for clinical trials and has performed well in the treatment of COVID‐19‐related multisystem inflammatory syndrome in children.^[^ ^141^ ^]^

However, there are several challenges with MSC therapies for inflammatory injury in laboratory and clinical research, including dose differences, pro‐inflammatory differentiation, and insufficient sources of high‐quality young donors.^[^ ^142^, ^143^, ^144^, ^145^, ^146^ ^]^ Lots of studies found that MSC‐EVs also have outstanding therapeutic potential and application value, and may be one of the important means to address the above challenges. In addition to their low immunogenicity, MSC‐secreted EVs present multiple anti‐inflammatory factors and growth factors on their membranes, and exhibit a long half‐life in vivo and non‐differentiating capacity, allowing the administration of higher doses for rapid inflammation suppression and damage repair.^[^ ^147^, ^148^, ^149^ ^]^ In a SARS‐CoV‐2‐induced acute lung injury (ALI) model, MSC‐EVs effectively reduced the inflammatory response, restored ACE2 activity on the surface of target cell membranes, and alleviated lung dysfunction.^[^ ^150^ ^]^ Synergistic treatment of MSC‐EVs, hydroxychloroquine, and azithromycin significantly reduced pro‐inflammatory markers, including neutrophil counts and D‐dimers, in patients with severe COVID‐19, resulting in the recovery of 71% of patients.^[^ ^151^ ^]^ In cellular infection assays, MSC‐EVs also inhibit SARS‐CoV‐2 replication,^[^ ^152^ ^]^ which may be related to intrinsically expressed IFN‐stimulated genes (ISGs) in MSCs.^[^ ^153^ ^]^ Moreover, inhalation treatment with MSC‐EVs in lipopolysaccharides (LPS)‐induced ALI model effectively reduced the expression of pro‐inflammatory factors, decreased the ALI pathological rating, promoted the polarization of macrophages toward the M2 subtype, and affected the expression of immune and redox mediators, such as toll‐like receptor (TLR) 4, arginase 1 (ARG1), heme oxygenase 1 (HO‐1), and nuclear factor erythroid 2‐related factor 2 (NRF2).^[^ ^154^ ^]^ Intrathecal injection of MSC‐EVs also effectively inhibited the activity of NLRP3 inflammasomes and associated TLR4/nuclear factor‐kappa B (NF‐kB) pathway in an interstitial cystitis model.^[^ ^155^ ^]^ Importantly, MSC‐EVs exert significant vascular healing and pro‐angiogenic effects in ischemic injury,^[^ ^156^, ^157^, ^158^ ^]^ which are potentially related to intrinsic miRNAs, including miR‐486‐5p and miR‐612, which promote VEGF expression.^[^ ^157^, ^158^ ^]^ This effect can be enhanced by hypoxia or pretreatment of MSCs with NO before harvest.^[^ ^157^, ^158^, ^159^ ^]^ MFGE8‐rich MSC‐EVs also aid in cardiac repair by promoting phagocytosis and the clearance of dead cardiomyocytes by immune cells.^[^ ^160^ ^]^ All the above studies imply the potential of MSC‐EVs in the treatment of the COVID‐19‐induced cytokine storm and tissue damage, particularly pulmonary fibrosis.^[^ ^161^, ^162^ ^]^ Admittedly, other types of EVs, such as those from fibroblasts, may also have therapeutic potential after engineered modification and can be further explored in future studies.^[^ ^163^ ^]^ However, in terms of immunogenicity and natural therapeutic potential, MSCs are still the preferred cell source for engineered vesicle design and preparation in the industry. Several ongoing clinical trials using MSC‐EV for the treatment of COVID‐19 include NCT04276987, NCT04491240, NCT04798716, NCT05216562, NCT05354141, NCT05125562, ChiCTR2000030261, and ChiCTR2000030484.

However, for the time being, it still should be prudent to consider the large‐scale introduction of MSC‐EVs into COVID‐19 clinical treatment for several reasons. First, the detailed mechanism of MSC‐EV therapy for COVID‐19 remains unclear, and numerous studies are required to elucidate both the important molecular components that exert benefits and toxic cargos that may induce side effects in MSC‐EV treatment. Moreover, strict standardization of the source and purification process of the MSC‐EVs is required for their clinical application.^[^ ^164^ ^]^ Compared with MSC‐EVs derived from young donors, aging MSC‐EVs fail to alleviate LPS‐induced ALI, reduce macrophage recruitment, and alter macrophage phenotypes, which is associated with differences in macrophage polarization‐related miRNA component preferences and internalization of MSC‐EVs.^[^ ^165^ ^]^ Adipose‐derived MSC‐EVs are more likely to increase thrombosis risk than bone marrow‐derived MSC‐EVs because of their higher expression of phosphatidylserine and TF.^[^ ^166^, ^167^ ^]^ These identified and unidentified biotoxicity, compositional differences, and effectors are potential threats to the safety of MSC‐EVs applications that must be clearly explored and adequately addressed before MSC‐EVs can be used in clinical treatment. International Society for EVs and International Society for Cell and Gene Therapy also strongly urge investigators and developers to carefully weigh the potential benefits and risks of using MSC‐EVs for COVID‐19 by obtaining rigorous and reliable laboratory and clinical data, and carefully evaluate the use of EVs through sound clinical trial design using well‐characterized EV formulations manufactured under strict good manufacturing practices (GMP) conditions and appropriate regulatory oversight.^[^ ^164^ ^]^

## 5 Engineering EVs as Drug Deliverers, Vaccines, and Vesicle Traps for COVID‐19

The endogenous functions of MSC‐EVs, such as inhibition of inflammation and damage repair, make them popular, however, these functions also require stringency in production quality control and mechanism exploration, leading to greater time and human and material costs for the development and clinical application of MSC‐EV therapies. In the face of rapidly developing novel viral pandemics, an effective integrated strategy for pandemic control is the use of EVs as editable nanovectors to deliver small molecule drugs or miRNAs for therapeutic purposes, deliver viral antigens as vaccines, or load virus‐bound host receptors as neutralizing traps.

First, EVs are derived from natural cells and exhibit better biocompatibility. Compared to synthetic lipid nanoparticles (LNPs), they can effectively avoid macrophage‐mediated immune clearance without introducing the allergenic safety risks of PEG. Polyethylene glycol (PEG) is one of the clinically approved modification strategies to reduce the absorption of nanoparticles by the immune system, which can reduce the interaction between nanoparticles and cells. However, while weakening their binding with immune cells, PEG also reduces LNPs uptake by target cells. More importantly, PEG nanoparticles may activate the complement system in patients, which carries the risk of triggering hypersensitivity reactions.^[^ ^168^ ^]^ While EVs derived from natural cells can effectively avoid such risks and side effects.^[^ ^169^ ^]^ Second, regarding drug loading, incubating the isolated and purified EVs with small molecule drugs allows their loading with these drugs, and the targeting effect of specific EVs can greatly reduce the required blood concentration for the same therapeutic effect.^[^ ^170^, ^171^, ^172^ ^]^ Moreover, parent cells may be genetically edited via genetic engineering methods including transfection, lentiviral infection, and CRISPR, thus producing numerous EVs enriched with therapeutic biomolecules.^[^ ^173^ ^]^ Therefore, from the COVID‐19 pandemic outbreak to the present, there have been increasing proposals and studies using EVs to deliver targeted potent drugs, design vaccines, and design neutralization traps.

### 5.1 Small‐Molecule Antiviral Prodrugs and Exosomal miRNAs against SARS‐CoV‐2

**Figure 4:**
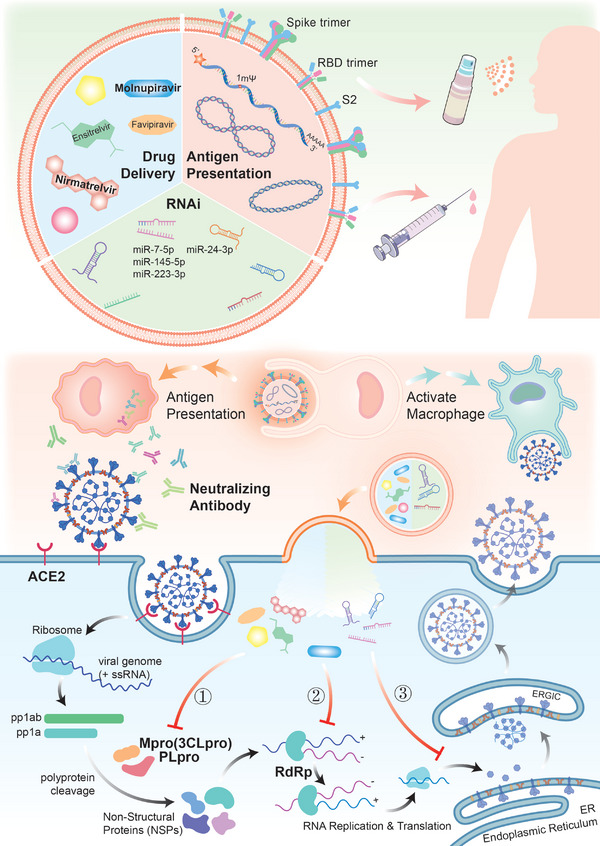
Engineered EV‐based targeted drug delivery and COVID‐19 vaccines. Expression of SARS‐CoV‐2 antigens (including spike protein trimer, receptor binding domain [RBD] trimer, and S2 domain) on the EV membrane surface or encapsulation of mRNA expressing antigens with EVs as nanovaccines. EV‐nanovaccine injection activates immunity and promotes the synthesis of specific neutralizing antibodies. EV‐encapsulated small molecule drugs or miRNAs are delivered to infected recipient cells and then specifically inhibit three core steps of viral proliferation: 1) main protease (Mpro)‐ and papain‐like protease (PLpro)‐mediated multiprotein hydrolysis and production of non‐structural proteins; 2) RNA‐dependent RNA polymerase (RdRp)‐mediated replication of the viral genome; and 3) translation and assembly of viral structural proteins.

After SARS‐CoV‐2 infects host cells, the viral genome (positive‐sense single‐stranded RNA) released into the cytoplasm will bind to the host ribosome and be translated into two polyproteins, pp1ab and pp1a.^[^ ^174^, ^175^ ^]^ Subsequently, the virus‐derived protein hydrolases, main protease (Mpro) and papain‐like protease (PLpro), precisely cleave pp1ab and pp1a to produce various mature nonstructural proteins (nsp1‐16) to perform functions including assisting viral replication and suppressing host antiviral immunity.^[^ ^176^, ^177^, ^178^, ^179^ ^]^ Among them, nsp12 acts as an RNA‐dependent RNA polymerase (RdRp) in combination with nsp7/nsp8 to execute the replication of the viral genome.^[^ ^180^, ^181^ ^]^ After the translation of structural proteins S, E, M, and N, and the assembly of viral particles at the endoplasmic reticulum‐Golgi intermediate compartment (ERGIC), the offspring viruses are released outside the cell via the host vesicle network to complete the viral replication cycle.^[^ ^181^ ^]^ Mpro, PLpro, and RdRb perform crucial roles in the viral replication process, which demonstrates why the coding sequences of Mpro and PLpro hydrolases and RdRb are highly conserved in the continuously added SARS‐CoV‐2 mutant strains.^[^ ^177^, ^182^ ^]^ And they are also the key targets of the antiviral drugs currently being developed (**Figure** **4**).^[^ ^178^, ^179^, ^183^, ^184^, ^185^, ^186^ ^]^

Nirmatrelvir (PF‐07321332) is a representative inhibitor of Mpro hydrolase and the core component of Paxlovid, one of the only two small‐molecule oral anti‐SARS‐CoV‐2 drugs that have successfully passed clinical trials and been approved for marketing.^[^ ^185^, ^186^ ^]^ However, due to the high rate of hepatic metabolism of Nirmatrelvir, Pfizer was forced to add Ritonavir into Paxlovid to inhibit the activity of the cytochrome enzyme CYP3A4 to maintain the blood concentration of Nirmatrelvir.^[^ ^187^ ^]^ Another FDA‐approved oral prodrug is Molnupiravir, a nucleoside antiviral that is taken up by RdRp and incorporated into the viral genome to induce lethal mutagenesis.^[^ ^188^ ^]^ However, Molnupiravir may be metabolized by the patient's normal cells and incorporated into the host genome leading to the risk of mutagenesis.^[^ ^189^ ^]^ Based on current clinical data, both drugs have performed well in targeting COVID‐19 caused by multiple prevalent variants and reducing the development of severe critical illnesses. However, issues of drug metabolism and targeting limit their dosing strategy.^[^ ^187^, ^190^, ^191^, ^192^ ^]^ Additionally, oral small molecule prodrugs that are still in clinical trials, such as Ensitrelvir (s217622), PF‐07304814, ALG‐097111 targeting Mpro,^[^ ^193^, ^194^, ^195^ ^]^ GRL0617, YM155, XR8‐23/24 targeting PLpro,^[^ ^196^, ^197^, ^198^ ^]^ and Remdesivir, Favipiravir, and Galidesivir targeting RdRp,^[^ ^199^ ^]^ are expected to perform well in the future, however, consideration of the related efficacy, safety, and pharmacokinetic issues is necessary. It is a feasible strategy to address the above issues through establishing a standardized EV‐based drug delivery platform. Although EVs have not yet been used to deliver prodrugs in COVID‐19‐related therapeutics, they have been assessed in the treatment of inflammatory diseases and cancer. For example, local delivery of curcumin‐albumin‐EVs using dissolvable microneedle arrays (dMNAs) may effectively inhibit and reverse LPS‐induced skin inflammation in mice and rats.^[^ ^200^ ^]^ Furthermore, injection of curcumin‐encapsulated EVs into an LPS‐induced septic shock mouse model via the tail vein improves the stability and targeting of curcumin and significantly enhances the anti‐inflammatory effects of curcumin.^[^ ^201^ ^]^ Doxorubicin (DOX)‐loaded tumor EVs induce effective tumor suppression in both subcutaneous and orthotopic glioblastoma models in mice.^[^ ^202^, ^203^ ^]^ Currently, high loading efficiency is typically achieved via passive incubation for the encapsulation of hydrophobic small molecule drugs in EVs. Conversely, for hydrophilic drugs, the use of saponin treatment, mild sonication, or electroporation is optional to improve the permeability of EV membranes.^[^ ^171^, ^204^, ^205^ ^]^ Alternatively, the natural inflammatory tendencies of MSC‐EVs or platelet‐derived EVs can be exploited to increase the targeting of anti‐SARS‐CoV‐2 prodrugs loaded in EVs to infected endothelial and immune cells. Moreover, expressing a receptor‐binding domain (RBD) on the membrane surface of EVs may achieve specific chemotaxis and drug presentation of EVs in target cells and tissues that highly express ACE2.^[^ ^173^ ^]^

In addition to using EVs to deliver prodrugs that target key viral proteases, administering human circulating exosomal miRNAs may directly intervene with viral replication. For instance, high‐throughput sequencing screening and validation of viral infection experiments revealed that four miRNAs (miR‐7‐5p, miR‐24‐3p, miR‐145‐5p, and miR‐223‐3p), which are more abundant in serum exosomes of young adults than in elderly and diabetic patients, directly inhibit viral replication and S protein expression.^[^ ^206^ ^]^ Bioinformatic prediction of human miRNAs and experimental validation identified five miRNAs (miR‐219a‐2‐3p, miR‐30c‐5p, miR‐378d, miR‐29a‐3p, and miR‐15b‐5p) that interfere with viral replication, and miR‐15b also inhibits the expression of S protein.^[^ ^207^ ^]^ Interestingly, exosomal miRNAs from some plants also inhibit viral replication and virus‐induced lung inflammation. For example, miR2911 enriched in the EVs from honeysuckle effectively inhibits viral replication in patients after oral absorption and improves the seven‐day negative conversion rate.^[^ ^208^ ^]^ And the ginger exosomal miRNAs aly‐miR396a‐5p and rlcv‐miR‐rL1‐28‐3p inhibit the expression of viral nsp12 and S proteins and abolish nsp12/nsp13‐mediated lung inflammation in mice.^[^ ^209^ ^]^

### 5.2 EV‐based SARS‐CoV‐2 Antigen Delivery as Vaccines

In the face of pathogenic microorganisms with extremely high transmission efficiencies, such as SARS‐CoV‐2, the most rapid and effective means of prevention and control is vaccination. This pre‐evokes the host immune response against the virus, including the synthesis of specific antibodies and activation of immune lymphocytes, by introducing non‐toxic viral surface antigens or live attenuated vaccines into the host (Figure 4). The SARS‐CoV‐2 mRNA vaccines, represented by Pfizer's BNT162b2 and Moderna's mRNA‐1273, have been urgently licensed and widely administered, and have demonstrated excellent prophylactic efficacy against COVID‐19 worldwide.^[^ ^210^, ^211^, ^212^, ^213^ ^]^ The current industrialized vector for delivering mRNA encoding the full‐length spike protein is lipid nanoparticles (LNPs), and EVs loaded with mRNA encoding S and N proteins also induce long‐term cellular and humoral immune responses in mice and effectively avoid the cytotoxicity of LNPs.^[^ ^214^ ^]^ The inhalation of a dry powder of mRNA vaccine composed of human lung‐derived EVs exhibits greater distribution to the bronchioles and parenchyma than standard synthetic nanoparticle liposomes in both mice and the African green monkey and elicits greater antigen‐specific IgG and IgA responses.^[^ ^215^ ^]^ However, because the linear mRNA encoding the full‐length S protein is too long and thus less stable, some researchers have designed a circular RNA vaccine encoding the trimeric RBD of S protein and confirmed that this vaccine elicits potent neutralizing antibodies and T cell responses in both mice and rhesus macaques.^[^ ^216^ ^]^

Injection of antigenic proteins or peptides as recombinant protein vaccines directly induces antiviral immune responses. For example, Novavax's recombinant protein vaccine NVX‐CoV2373, which uses the S protein trimer as a direct antigen, achieved a protection rate of 90.4% in phase III clinical trials.^[^ ^217^, ^218^, ^219^, ^220^ ^]^ However, the exposed portion and spatial array distribution of the S protein in recombinant protein vaccines are not identical to those on SARS‐CoV‐2, and thus, may induce non‐specific neutralizing antibodies that are ineffective against SARS‐CoV‐2.^[^ ^221^ ^]^ Using EV membranes that are homologous to the viral envelope as display platforms for antigenic proteins may lead to improved neutralizing antibody induction.^[^ ^222^ ^]^ For example, a comparative study prior to the COVID‐19 pandemic designed an EV‐vaccine that expressed the S protein of SARS and induced high levels of specific neutralizing antibodies in mice.^[^ ^223^ ^]^ The RBD of the SARS‐CoV‐2 S protein, as the central site for direct interaction with the host receptor ACE2, has received attention from vaccine design developers. An inhalable lyophilized vaccine composed of recombinant SARS‐CoV‐2 RBD conjugated to lung‐derived EVs elicits the synthesis of RBD‐specific IgG antibodies and activation of Th1‐like CD4+ and CD8+ T cells in the lungs of mice, effectively alleviating the lung inflammation caused by SARS‐CoV‐2 infection. In this study, researchers derived lung spheroid cells (LSCs) whose regenerative abilities have been demonstrated from human lung donor samples and purified LSC‐EVs from human LSC‐secretome via 100 kDa ultrafiltration. The commercial recombinant SARS‐CoV‐2 RBD protein was subsequently conjugated to LSC‐EV using a DSPE‐PEG‐NHS linker (1,2‐Distearoyl‐sn‐glycero‐3‐phosphoethanolamine‐poly(ethylene‐glycol)‐N‐hydroxysuccinimide) to obtain the final inhaled vaccine RBD‐EVs.^[^ ^224^ ^]^ To further improve the immune activation of RDB‐EV vaccines, some researchers have isolated and purified bacterial EVs from the culture supernatant of *Salmonella typhimurium* genetically expressing SpyCatcher peptide by high‐speed centrifugation. These bacterial EVs have a large number of SpyCatcher peptides embedded on their membrane surface. Then the recombinant His‐Spy‐RBD proteins were eukaryotically purified from 293F cells. The coupling of the bacterial EV membrane surface with RBD was achieved by covalent crosslinking of the amide bonds of the SpyTag/SpyCatcher system. After intranasal inoculation, this bacteria‐derived RBD‐EV rapidly drives inflammatory responses and effectively activates immune cells including dendritic, T, and B cells by endotoxin‐mediated immunostimulatory properties.^[^ ^225^ ^]^ In addition to using RBD‐EV to induce host‐neutralizing antibody production, other researchers have also induced a strong and specific CD8+ T‐cell immune response by EV‐delivery of T‐cell epitope peptides against SARS‐CoV‐2 in HLA‐A transgenic mice. They harvested red blood cells (RBCs) from blood samples of healthy donors and isolated RBC‐EVs from culture supernatants by differential ultracentrifugation, and then coupled 31 directly synthesized T‐cell epitope peptides on the membrane surface of RBC‐EVs using the same DSPE‐PEG‐NHS linker. Ultimately, they obtained these EV vaccines with specific T cell immune activation.^[^ ^226^ ^]^ Moreover, due to the increasing SARS‐CoV‐2 variants and multiple‐site mutations on the S protein that may mediate immune escape, relatively conserved subunits of S protein, such as the heptad repeat (HR) and S2 subunits, have been added to the RBD‐nanoparticle vaccines. This elicits more robust neutralizing antibody‐dependent cytotoxicity and effective cross‐protection responses against multiple variants.^[^ ^227^, ^228^ ^]^ Such design strategies should also be considered in future EV‐vaccine development.

### 5.3 ACE2‐EVs as Neutralization Traps for SARS‐CoV‐2 Infection

Currently, another major therapeutic strategy for SARS‐CoV‐2 infections is blocking further viral infiltration of host cells by exogenous injection of neutralizing antibodies into patients. However, with the increasing number of SARS‐CoV‐2 variants, variants of concern (VOCs), specifically Omicron, have shown increasing immune escape capacity by increasing key mutations in their S proteins (e.g., D614G, N501Y, K417N, and E484A). This is evidenced by reduced binding to most neutralizing antibodies (including monoclonal antibody drugs and vaccine‐induced neutralizing antibodies) and increased affinity for the major receptor ACE2.^[^ ^229^, ^230^, ^231^ ^]^ This poses a great challenge to the neutralization and blocking strategy. The introduction of exogenous ACE2 protein competitively binds to the SARS‐CoV‐2 S protein, blocking interaction with ACE2 on the host cell membrane surface, and this blocking therapeutic modality has shown corresponding advantages. Human recombinant soluble ACE2 (hrsACE2) and recombinant ACE2 fusion proteins (e.g., hACE2‐Fc and hACE2‐Ig) effectively neutralize wild‐type SARS‐CoV‐2 and multiple variants with enhanced infectivity, demonstrating their potent and broad‐spectrum antiviral effects.^[^ ^232^, ^233^, ^234^, ^235^ ^]^

However, some concerns with the soluble ACE2 and recombinant ACE2 fusion proteins cannot be ignored. First of all, high titers are necessary for soluble ACE2 and ACE2‐Fc fusion proteins to block SARS‐CoV‐2 invasion because they are small single protein molecules with small spatial size, whereas viral particles are large and have amount of spike proteins embedded on the envelope surface. But it is pharmacokinetic difficult to achieve such a high titer objectively. Although Fc fusion strategy can improve the pharmacokinetics of ACE2 by extending the half‐life of free ACE2 and making the purification of recombinant proteins more convenient, ACE2‐IgG‐Fc may cause antibody‐dependent enhancement (ADE) during viral infection. In other words, after ACE2 terminal of ACE2‐IgG‐Fc binding with spike of SARS‐CoV‐2, the Fc terminal may bind with FcR of some host cells, which promotes viral invasion and lead to harmful inflammatory and tissue damage. The ACE2 multimers based on protein scaffolds effectively avoid the above‐mentioned immunotoxic, but their large molecular weight and rigid structure pose more stringent requirements on the purification process, and greatly increase the aggregation tendency and immune clearance of ACE2 recombinant proteins in vivo. EVs, as natural nano‐carriers that are abundantly present in the body, can provide a safer delivery mode for ACE2 neutralization strategies. First, they can significantly improve the in vivo half‐life of exogenous ACE2 due to the low immunogenicity of EVs. Second, EVs are similar in size to viral particles and can form a huge steric hindrance when bind with SARS‐CoV‐2, effectively blocking virus infection. More importantly, engineered EVs can provide a sufficient membrane display platform for ACE2. By enriching high‐abundance ACE2 on the EV membrane, high titer introduction of ACE2 and further multiplicity optimization can be achieved while avoiding aggregation and immune clearance.

**Figure 5:**
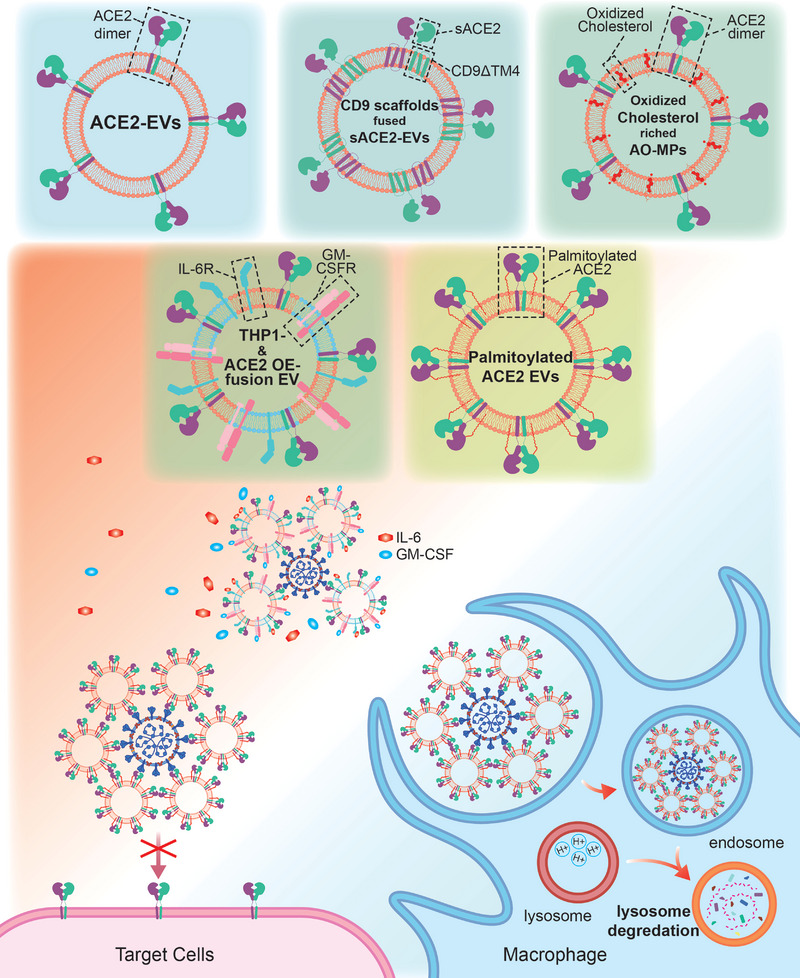
ACE2‐EVs as neutralizing traps for SARS‐CoV‐2 infection. Based on normal ACE2‐EVs, a variety of engineered ACE2‐EVs with different properties have been developed. Different methods have been used, including increasing the membrane ACE2 level and neutralization efficiency of EVs using palmitoylation modification or CD9 scaffolds, using oxidized cholesterol‐rich ACE2‐EVs to host SARS‐CoV‐2 to enhance lysosomal degradation efficiency in macrophages, and fusing THP‐1‐derived EVs and ACE2‐EVs which neutralize both SARS‐CoV‐2 and inflammatory IL‐6 and granulocyte‐macrophage colony‐stimulating factor (GM‐CSF) to rapidly alleviate the inflammatory storm. SARS‐CoV‐2 quarantined by ACE2‐EVs can no longer bind to the ACE2 receptor on the cell membrane and is then phagocytosed and degraded by macrophages. AO‐MP, ACE2‐overexpressing microparticles; sACE2, soluble ACE2.

Fortunately, high‐speed atomic force microscopy experiments have shown that EVs secreted by the ACE2‐expressing cell line PC‐9 also bind directly to SARS‐CoV‐2 particles and initiate the membrane docking mechanism via S‐ACE2 interaction, suggesting the feasibility of using ACE2‐EVs as neutralization traps for SARS‐CoV‐2 infection.^[^ ^236^ ^]^ ACE2‐EVs from the plasma of patients with COVID‐19 and ACE2‐EVs secreted by the ACE2‐expressing human lung epithelial cell line Calu‐3 also block SARS‐CoV‐2 and VOC infections more efficiently than soluble ACE2 in vitro.^[^ ^237^, ^238^, ^239^ ^]^ Both human lung spheroids cell‐derived ACE2‐EVs and plasma‐derived ACE2‐EVs from patients with COVID‐19 delivered via inhalation therapy promote viral clearance and reduce lung injury in cynomolgus macaques and hACE2 transgenic mice challenged with authentic SARS‐CoV‐2 (**Figure** **5**).^[^ ^237^, ^240^ ^]^ Significantly, the level of ACE2 on the ACE2‐EV surface is positively correlated with its ability to block SARS‐CoV‐2,^[^ ^238^ ^]^ and EVs containing high levels of the viral receptor ACE2 in bronchioalveolar lavage fluid (BALF) from critically ill patients with COVID‐19 are associated with reduced intensive care unit (ICU) and hospitalization times.^[^ ^241^ ^]^ Therefore, some efforts have been made to increase the ACE2 content on the surface of ACE2‐EV membranes. Researchers have increased ACE2 expression in human bronchial epithelial cells 16HBE and H1299 using IFN*α*/*β* treatment, thereby increasing the ACE2 content in EVs.^[^ ^242^ ^]^ Moreover, fusing the S‐palmitoylation‐dependent plasma membrane (PM) targeting sequence with the ACE2 protein promotes the membrane targeting and EV secretion of exogenously expressed ACE2 in cells. Both an in vitro infection assay and authentic SARS‐CoV‐2 challenge in hACE2 transgenic mice also showed that PM‐ACE2‐EV at a low dose prevents viral infection and reduces lung injury.^[^ ^59^ ^]^ As CD9 is an essential EV surface tetraspanin, truncated CD9 scaffolds have been designed to display soluble ACE2 (sACE2) variants with enhanced affinity for S protein on the EV surface, and viral infection assays verified the blocking effect of these engineered ACE2‐EVs on wild‐type SARS‐CoV‐2 and its variants.^[^ ^243^ ^]^ Additionally, it is pleasing to note that while we were conducting this review, Eugene M Obeng et.al. also noted and carefully addressed the importance of multivalency in ACE2 nanomedicine engineering, as well as novel approaches for developing and implementing multivalent therapies.^[^ ^244^ ^]^

Interestingly, researchers have increased the function of ACE2‐EVs by introducing additional components. EVs obtained using the A549 cell line overexpressing ACE2 were enriched in oxidized cholesterol, which increases the endosomal pH and decreases the lysosomal pH in alveolar macrophages that uptake ACE2‐EVs, thereby promoting lysosomal degradation of ACE2‐EV‐bound SARS‐CoV‐2 in macrophages.^[^ ^245^ ^]^ A novel decoy nanoparticle has been designed by fusing human monocyte‐derived EVs and ACE2‐expressing engineered cell‐derived EVs, which neutralize SARS‐CoV‐2 via ACE2 proteins. Simultaneously, this nanoparticle effectively neutralizes IL‐6 and granulocyte‐macrophage colony‐stimulating factor (GM‐CSF) via abundant cytokine receptors on its membrane surface derived from monocytes, thereby rapidly alleviating the inflammatory storm and reducing lung injury.^[^ ^246^ ^]^

The use of receptor‐EVs as neutralizing traps to block viral infestation is a practical and relatively simple design logic for a therapeutic strategy. During the pre/mid‐pandemic period in which new viruses emerge and spread rapidly but the targeted agents that inhibit viral amplification have not yet been screened, receptor‐EV formulations that are rapidly produced in systematic industrial plants can be used to counter virus transmission and mutations. However, before the systematic receptor‐EV production system is established, research societies and regulatory agencies need to establish standards and urge researchers, developers, and commercial companies to provide sufficient reliable laboratory and clinical data to demonstrate the security and efficacy of receptor‐EV formulations. Furthermore, the following issues should be considered and addressed: 1) whether the introduction of several relevant receptors causes physiological damage to patients; 2) the safety of EV carriers, for example, some tumor cell‐derived EVs may promote thrombosis;^[^ ^247^, ^248^ ^]^ and 3) the mode of administration of receptor‐EVs. For viruses that are mainly transmitted through the respiratory tract, such as SARS‐CoV‐2, receptor‐EVs may be administered via inhalation. However, for viruses that are transmitted by other means, such as human immunodeficiency (HIV) and rabies viruses, the feasibility of intravenous or intramuscular administration of receptor‐EVs and their half‐lives in the host also requires critical evaluation.

## 6 Perspective

As important transmitters of intercellular information, EVs systematically regulate the physiological state of the host through continuous proximal and distal transmission of proteins, lipids, nucleic acids, and various metabolites. Therefore, EVs have been increasingly used as a new generation of clinical diagnostic indicators to determine the progression of human diseases including cancers and immune inflammation. Moreover, they are potentially outstanding medical biologics and drug delivery nanocarriers because of their low immunogenicity, easy editing properties, and ability to fully inherit several medical functions from parent cells, particularly MSCs. In particular, the therapeutic strategy of receptor‐EV agents that block viruses, which emerged during the COVID‐19 pandemic, may also be used to rapidly respond to the emergence of new influenza viruses in the future.

The mainstream diagnostic approaches for patients infected with SARS‐CoV‐2 are nucleic acid amplification tests (NAATs) and antigen/antibody tests. However, the disease progression in patients, including asymptomatic infection, those patients with mild COVID‐19 disease who recover spontaneously, and those who are critically ill and require medical intervention, is unpredictable until the patients develop the appropriate clinical symptoms, which often results in unnecessary loss of medical resources and missed optimal treatment. EVs, as forerunners in the characterization of tissue and organ status, have the potential to be developed as indicators to assist in the diagnosis and prediction of COVID‐19. TF, COPB2, IL‐18R1 in peripheral blood EVs, activated caspase‐3, and MLKL in platelet EVs exhibit strong correlations with the risk of microthrombosis, malignant disease progression, and hospital stay in patients with COVID‐19. These above indicators, as well as new indicators under development, can be combined to predict and classify patients with COVID‐19 to rationally allocate medical resources.

A major technical challenge limiting the use of EVs in disease diagnosis is the establishment of clinically applicable standardized EV isolation, purification, and detection systems. Current commercial EV isolation kits enable efficient sample processing and high reproducibility of body fluid samples, including blood, plasma, urine, and saliva. A major class of commercial kits utilizes proprietary precipitation reagents that alter the solubility of the biological fluid, thus inducing precipitation of poorly soluble fractions, including EVs, after low‐speed centrifugation. The purity of EVs obtained using this separation technique is low and scarcely meets the criteria for disease diagnosis and development prediction. Another class of commercial kits is pre‐packaged columns with designs based on size‐exclusion chromatography. These columns rapidly separate and purify high‐purity EVs from patient sample solutions and fit well into automated detection platforms. However, reusing separation columns poses the risk of sample contamination and the cost of disposable columns is high. Using multiple separation columns to process samples in parallel or reducing the cost of column packing may be viable solutions. In contrast, microfluidic chips currently have more potential than columns for eventual application in clinical sample EV automation platforms. Using nanoscale size channels, laminar flow, and applied physical fields, microfluidic chips precisely manipulate microscale fluids and separate particles of different sizes without introducing contaminant sources. This efficiently performs high‐precision separation and sensitivity detection in clinical samples with limited volume.^[^ ^249^, ^250^ ^]^ Despite the relatively demanding manufacturing process of microfluidic chips, their extremely low contamination and feasible reusability make them the most suitable EV separation and detection technology for clinical diagnostic EV automated analysis platforms.

EV medical agents used for clinical treatment, including MSC‐EVs, drug‐EV delivery systems, engineered EV‐based vaccines, and neutralization traps, present challenges. The first is the choice of the best EV source. MSCs are currently the most promising source, as several studies indicate that MSC‐EVs modulate inflammation and repair and regenerate damaged tissues.^[^ ^251^, ^252^, ^253^ ^]^ In contrast, if tumor cells are selected as the parent cells for expanded culture and mass production of EVs, the tumor‐derived EVs (TEVs) may inherit potentially oncogenic characteristics and pose a risk of promoting cancer development after high‐dose administration of TEVs in patients. Exploring and developing methods to deactivate or remove unwanted and harmful EV content may be an important and novel engineering strategy to overcome the medical limitations of TEVs.^[^ ^254^ ^]^ Furthermore, despite the lack of biotoxicity in MSC‐EVs, the supply of high‐quality clinical‐grade human MSCs is inadequate in the face of the high demand for the use of EV medical preparations. Some solution strategies include immortalizing MSCs, establishing cell banks, using human induced pluripotent stem cells (iPSCs) to produce MSCs with limitless expandability, and increasing the yield of EVs by physical extrusion. However, further efforts are required to ensure the efficacy of these MSC‐EVs.^[^ ^251^, ^255^, ^256^, ^257^ ^]^ Platelet‐derived EVs are considered potential therapeutic agents for regenerative therapy because of their enrichment in growth factors (such as platelet‐derived growth factor, TGF‐*β*, and VEGF). Moreover, their natural inflammatory chemotaxis can be exploited for targeted delivery of anti‐inflammatory agents, but cannot be applied in inflammatory diseases with a risk of microthrombosis such as COVID‐19.^[^ ^258^ ^]^

Second, to maintain consistent EV production and homogeneity of the EV cargo, strict quality control measures are required to ensure that each step of EV production complies with the guidelines for drug manufacturing and clinical application proposed by regulatory agencies. The establishment of corresponding official monitoring platforms is necessary to regularly inspect the cell culture environment, production and modification processes, and characterization and storage of EV medical preparations from manufacturers.

Last, the dosage of EV formulations and their route of administration significantly affect their efficacy in clinical treatment, which are complex issues that researchers and dosing physicians must consider. For example, in several projects using MSC‐EV for the treatment of COVID‐19, researchers have typically converted the dosage of EV agents based on the cell dosage of MSC therapy, and these conversions clearly require further optimization, that is, the direct relationship between the dosage of EV agents and therapeutic effect should be fully explored. Moreover, in COVID‐19 animal experiments and clinical trials, intranasal administration has been commonly used because the primary therapeutic purpose was to suppress viral infection and inflammatory storms in the respiratory tract and lungs. Fortunately, EV agents can reside in the lungs for long periods of time. However, quantitative distribution research using fluorescent probe‐labeled EVs reveals that EVs are predominantly distributed in the liver, lungs, and spleen after intravenous injection, while intramuscularly injected EVs accumulate in the lymph.^[^ ^259^, ^260^, ^261^ ^]^ These properties require the development of engineered EV strategies that focus on EV pharmacokinetic and pharmacodynamic characteristics and improve the targeting of EV formulations to ensure that desired therapeutic effects are achieved without causing toxicity.

Overall, EVs demonstrate multiple potential effects in COVID‐19 diagnosis, treatment, and prevention, particularly in the use of ACE2‐EVs as a blocking strategy for SARS‐CoV‐2 neutralization traps, which provide important ideas for the initial prevention, control, and treatment of novel viruses. However, further research and efforts are required to realize the establishment of an automated platform for EV diagnosis, and the safe production and clinical application of EV medical agents.

## Conflict of Interest

The authors declare no conflict of interest.

## Author Contributions

P.S., Y.W., F.X., and Q.Z. contributed equally to this work. P.S. and Y.W. conceived and drafted the manuscript. P.S. and F.X. drew the figures. P.S. and Q.Z. collected the information. P.S., Y.W., F.X., Q.Z., L.C., and Z.L. discussed the concepts of the manuscript. X.M., F.Z., and L.Z. directed the manuscript.
